# Supplementary material for: Antimicrobial Dispensing Practice in Community Pharmacies in Russia during the COVID-19 Pandemic
Source: Antibiotics (Basel). 2022 Apr 27;11(5):586. doi: 10.3390/antibiotics11050586 (PMC9137661; doi:10.3390/antibiotics11050586)
Supplement: Supplementary file 1 [file antibiotics-11-00586-s001.zip › antibiotics-1702406-supplementary.pdf]

Supplementary Materials

Table S1. Most common AM agents supplied to different age groups, n (%) of total supplies

Group of AM	< 12 years	13-18 years	19-35 years	36-60 years	> 60 years	Total
**Antibiotics**						
Macrolides (J01FA)	37 (26.1)	9 (17.3)	156 (11.9)	394 (16.5)	104 (13.1)	700 (14.9)
Combinations of penicillins, including beta-lactamase inhibitors (J01CR)	37 (26.1)	12 (23.1)	162 (12.4)	276 (11.6)	89 (11.2)	576 (12.3)
Fluoroquinolones (J01MA)	5 (3.5)	4 (7.7)	121 (9.2)	304 (12.7)	101 (12.7)	535 (11.4)
Third-generation cephalosporins (J01DD)	20 (14.1)	2 (3.8)	91 (6.9)	155 (6.5)	71 (8.9)	339 (7.2)
Penicillins with extended spectrum (J01CA)	13 (9.2)	5 (9.6)	68 (5.2)	123 (5.2)	38 (4.8)	247 (5.3)
Tetracyclines (J01AA)	0	2 (3.8)	39 (3.0)	37 (1.6)	23 (2.9)	101 (2.2)
Second-generation cephalosporins (J01DC)	4 (2.8)	4 (7.7)	9 (0.7)	14 (0.6)	7 (0.9)	38 (0.9)
**Antivirals**						
Other antivirals (J05AX)	19 (13.4)	12 (23.1)	260 (19.8)	396 (16.6)	117 (14.7)	804 (17.2)
Nucleosides and nucleotides, excluding reverse transcriptase inhibitors (J05AB)	1 (0.7)	0	79 (6.0)	108 (4.5)	55 (6.9)	243 (5.2)
Cyclic amines (J05AC)	4 (2.8)	0	31 (2.4)	64 (2.7)	27 (3.4)	126 (2.7)
**Antifungals**						
Triazole derivatives (J02AC)	0	1 (1.9)	122 (9.3)	106 (4.4)	42 (5.3)	271 (5.8)
**Others***	2 (1.4)	1 (1.9)	172 (13.1)	410 (17.2)	122 (15.3)	707 (15.1)

Comment: *groups of AM agents accounted ≤3% of total supplies as for each group
